# Antibody-drug conjugates in colorectal cancer: current landscape and future perspectives from clinical trials

**DOI:** 10.3389/fonc.2026.1843037

**Published:** 2026-06-19

**Authors:** Jingyi Li, Yemin Xu, Lu Wang, Ying Zhu, Bin Deng

**Affiliations:** Department of Gastroenterology, Northern Jiangsu People's Hospital Affiliated to Yangzhou University, Yangzhou, China

**Keywords:** antibody-drug conjugate (ADC), clinical trials, colorectal cancer, landscape analysis, target therapy

## Abstract

**Background:**

Advanced colorectal cancer (CRC) has limited therapeutic options. Antibody-drug conjugates (ADCs) deliver cytotoxic agents selectively, minimizing systemic toxicity. This study outlines the current clinical trial landscape of ADCs in CRC to identify research gaps and future directions.

**Methods:**

We queried the Trialtrove database (up to August 2025) for non-observational trials of ADCs in CRC. Analytical indicators included temporal trends, geographic distribution, sponsor type, target antigens, and payloads.

**Results:**

Overall, 194 eligible trials were identified. Trial numbers have steadily increased since 2020, peaking in 2024. Most are open trials, predominantly conducted in China and the United States, and heavily industry-sponsored. HER2, Trop-2, and c-Met are the most frequently studied targets. Topoisomerase I inhibitors are the most common payloads, frequently paired with cleavable linkers. Crucially, the vast majority of these trials remain in early phases (Phase I/II), with Safety and tolerability remaining the primary endpoints.

**Conclusions:**

ADCs show preliminary therapeutic potential in CRC; however, assertions regarding broad efficacy must remain highly cautious given the early-stage, safety-oriented nature of the current pipeline. Furthermore, the current landscape faces challenges such as publication bias and industry dominance, which prioritize commercially viable hotspots over niche targets. Future progress relies on discovering novel targets, optimizing linker-payload designs, and exploring rational combination therapies.

## Introduction

Colorectal cancer (CRC) ranks among the most common malignant tumors worldwide, causing over 500,000 deaths annually and placing it among the top three cancers in terms of incidence and mortality rates (1). Although targeted therapies and immunotherapies have shown promising results in specific patient populations in recent years, treatment options remain limited for many patients, particularly those with advanced colorectal cancer. Antibody-drug conjugates (ADCs), as an innovative form of biotherapy, can selectively deliver cytotoxic agents to tumor cells, thereby minimizing systemic toxicity (2). Unlike previous narrative reviews, this study leverages the Trialtrove database to provide a comprehensive, quantitative landscape of 194 non-observational ADC clinical trials in CRC, encompassing trial phase, target antigens, payloads, sponsor types, and geographic distribution, thus offering a unique data-driven perspective on research trends and gaps. This study outlines the current status of clinical trials for ADC therapy in colorectal cancer, while exploring potential avenues for future research and clinical application strategies.

## Methods

We conducted a comprehensive search of theTrialtrove database (https://clinicalintelligence.citeline.com/), an authoritative clinical trial database integrating clinical trial data from dozens of countries and regions around the world, as of August 26, 2025, utilizing the retrieval strategy: “(Disease is Oncology: Colorectal) AND (Drug Type is Biological > Protein > Antibody > Antibody-drug conjugate) AND (Study Design does not contain Observational)”. Eligible trials were defined as non-observational ADC studies targeting CRC, regardless of trial phase. Observational studies and preclinical investigations were excluded. For multi-cohort or basket trials encompassing various solid tumors, only data specifically pertaining to the CRC cohorts were extracted and analyzed. Duplicate trials (e.g., multiple registrations for the same study) were identified and removed. Two independent researchers extracted, reviewed, and cross-checked all trial data—including unstructured records and ambiguous classification categories—to resolve any discrepancies, address information gaps, and mitigate classification bias. Where applicable, trial identifiers and tracking information were systematically cross-referenced with publicly available registries, such as ClinicalTrials.gov. To ensure methodological transparency and reproducibility, the detailed study selection process is illustrated in a PRISMA-style flow diagram (**Supplementary Figure S1**). Furthermore, strict classification criteria for target antigens, payloads, linker types, primary endpoints, and sponsor types were standardized prior to data extraction; the detailed definitions and categorization frameworks are provided in **Supplementary Table S1**. The comprehensive list and characteristics of the 194 included trials are provided in **Supplementary Table S2**. All clinical research-related charts in this study were based on raw clinical trial data retrieved from sources. Microsoft PowerPoint software was used for data visualization to construct the foundational framework of the charts. Subsequently, Adobe Illustrator software was employed for layout optimization, ultimately producing the standardized charts presented in the text.

## Results

**Figure 1:**
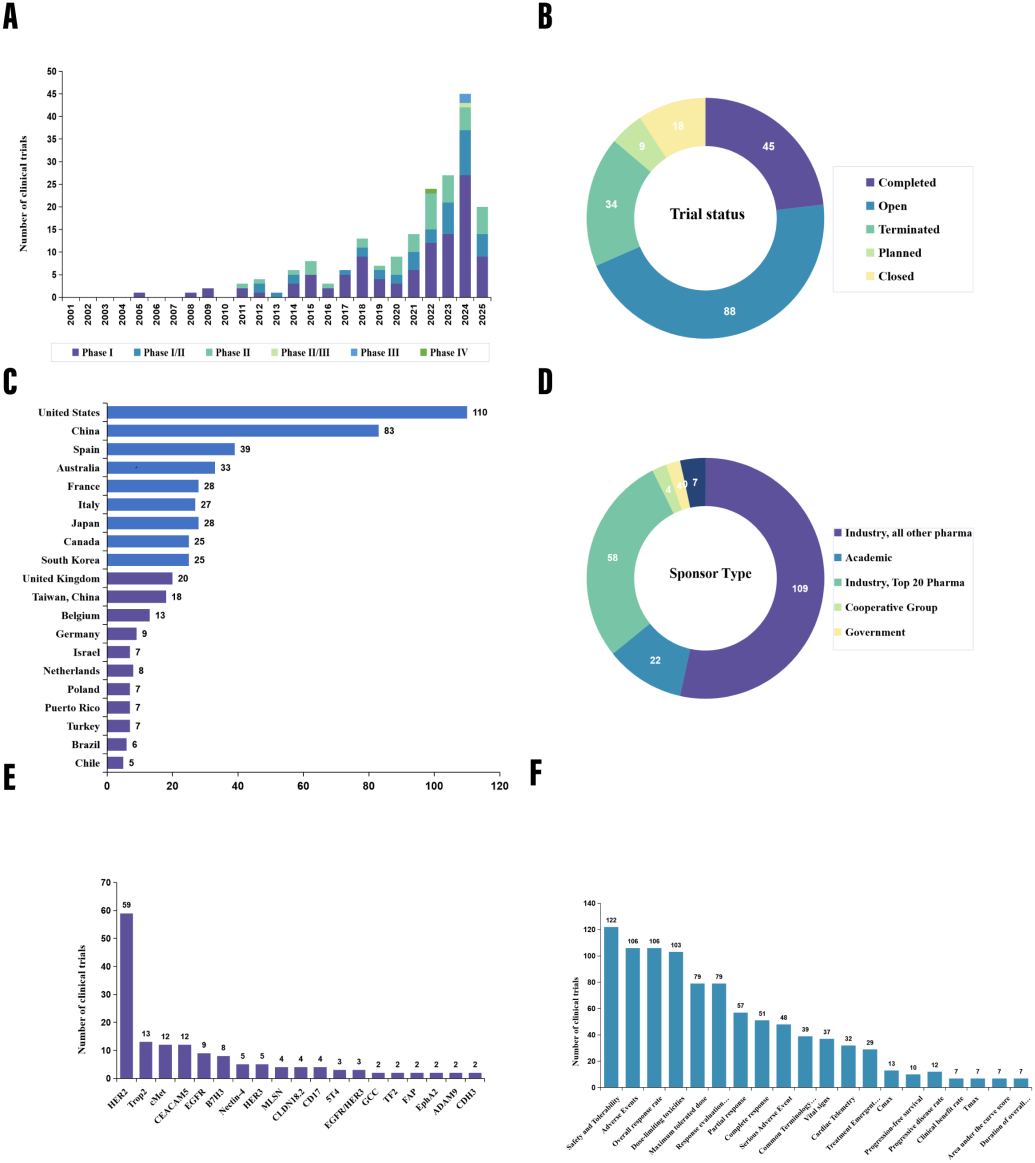
Comprehensive clinical trial landscape of antibody-drug conjugates (ADCs) in colorectal cancer (CRC) (N = 194). **(A)** Annual temporal distribution of ADC clinical trials from 2001 to 2025, stratified by development phase. The stacked bars illustrate a progressive surge in trial initiations since 2020, peaking in 2024, with a stark predominance of early-phase (Phase I and Phase I/II) evaluations. Data for 2025 reflects registrations up to the cutoff date of August 26, 2025. **(B)** Proportional distribution of trials by operational status, demonstrating a dynamic landscape led by active/open studies alongside notable attrition rates represented by terminated and withdrawn trials. **(C)** Geographic distribution mapping the top 20 countries and regions participating in CRC-directed ADC trials, dominated prominently by the United States (n = 110) and China (n = 83). **(D)** Breakdown of trial sponsor types. Funding is heavily skewed toward industry entities, divided between the top 20 global pharmaceutical companies (n = 58) and other commercial sponsors (n = 109), contrasted against a minor share from academic and cooperative groups. **(E)** Antigen target distribution of the top 20 evaluated biomarkers, highlighting extreme competitive saturation concentrated around HER2 (n = 59), Trop-2 (n = 13), and c-Met (n = 12). **(F)** Quantitative distribution of primary and secondary clinical trial endpoints. The prominent clustering of safety-related metrics (e.g., Safety and Tolerability, Adverse Events, and Dose-Limiting Toxicities) underscores that the global clinical pipeline is fundamentally safety-oriented. ADC, antibody-drug conjugate; CRC, colorectal cancer; CEA, carcinoembryonic antigen; CEACAM5, carcinoembryonic antigen-related cell adhesion molecule 5; CLDN18.2, claudin 18.2; EGFR, epidermal growth factor receptor; GCC, guanylyl cyclase C; HER2, human epidermal growth factor receptor 2; MLSN, mesothelin; TF2, tissue factor 2.

**Figure 2:**
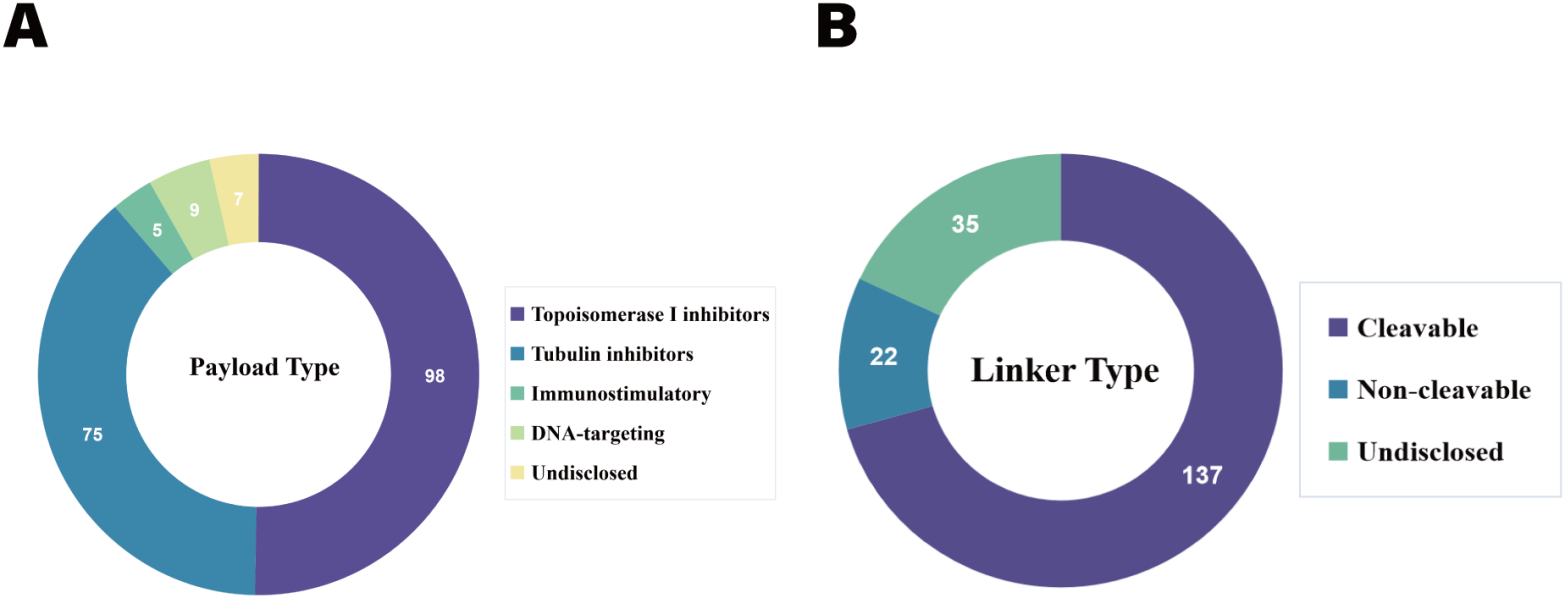
Distribution of cytotoxic payload mechanisms and chemical linker categories in CRC ADC clinical trials (N = 194). **(A)** Proportion and absolute counts of clinical trials stratified by the mechanism of action of the conjugated cytotoxic payloads. Topoisomerase I inhibitors (n = 98) and Tubulin inhibitors (n = 75) represent the foundational pillars of payload selections, while immunostimulatory (n = 5) and DNA-targeting (n = 9) mechanisms comprise emerging niches. **(B)** Proportional distribution of linkers classified by their biochemical release mechanism. Cleavable linkers (n = 137) are predominantly utilized across the pipeline to leverage physiological triggers for payload release and exploit the bystander effect, far exceeding non-cleavable configurations (n = 22). ADC, antibody-drug conjugate; CRC, colorectal cancer; DNA, deoxyribonucleic acid.

A total of 194 eligible trials were identified eventually. Temporal analysis showed an increase of trial number from 2001 to 2025. Compared to 2010–2019, clinical trials of ADC therapy for colorectal cancer have shown a steady upward trend since 2020, with trial numbers peaking in 2024 (**Figure 1A**). Crucially, stratification by trial phase reveals that this pipeline is heavily skewed toward early-stage clinical evaluation. Phase I and Phase I/II trials constitute the absolute majority of the historical and active landscape, whereas definitive, late-stage Phase III and Phase IV trials remain exceptionally rare, indicating that the field is still in its foundational expansion phase. In terms of operational progression, the identified studies are mainly in an open or active recruiting status (**Figure 1B**), reflecting vigorous and accelerating efforts in ADC innovation. However, a closer stratification of trial status indicates that a critical subset of trials has been terminated, suspended, or withdrawn. This subset reflects the inherent attrition rates within clinical development (**Figure 1B**). Notably, the explicit underlying reasons for individual trial closures—whether due to commercial strategic shifts, logistical challenges, safety signals, or efficacy outcomes—are frequently undisclosed or underreported in public registries, necessitating cautious interpretation regarding specific asset failures.Geographically, trials were predominantly conducted in China and the United States, which partially aligns with the variations in national disease burden and healthcare systems (**Figure 1C**). Furthermore, most studies were industry-sponsored, indicating strong commercial interest and market potential for ADC therapies (**Figure 1D**). Notably, further stratified analysis by sponsor type indicates that industry-sponsored trials are predominantly concentrated on advancing established hotspot targets (e.g., HER2) into later-stage development, whereas non-industry or academically sponsored trials more frequently explore novel combination strategies or niche populations. Regarding target antigens, HER2 is the most frequently studied, followed by Trop-2 and c-Met (**Figure 1E**). Target distribution exhibits high competitive saturation, with research heavily clustered around these top three established hotspots, while niche or emerging targets (e.g., Claudin 18.2 and CEA) represent only a minor fraction of the current clinical landscape. Regarding cytotoxic payloads, the most commonly used are topoisomerase I inhibitors (TOPIs), while tubulin inhibitors also account for a significant proportion (**Figure 2A**). Most ADCs employ cleavable linkers because they facilitate payload release and the bystander effect (**Figure 2B**). A temporal evaluation of ADC design strategies reveals that while TOPIs and traditional cleavable linkers remain historically dominant, there is an emerging shift toward proprietary, highly stable linkers and novel payload mechanisms in trials initiated after 2022. Finally, consistent with the early-phase dominance of the global pipeline, safety and tolerability were identified as the primary endpoint indicators across the majority of trials, far exceeding efficacy-driven survival measurements such as progression-free survival (PFS) or overall survival (OS) (**Figure 1F**). This distribution strongly underscores that the current ADC landscape in CRC is fundamentally safety-oriented, and large-scale, definitive efficacy conclusions cannot yet be widely drawn.

## Discussion

The disclosed clinical trial outcomes have demonstrated encouraging, albeit preliminary, safety profiles and initial signals of efficacy for ADCs in specific CRC cohorts. For instance, the ADC trastuzumab deruxtecan (T-DXd) exhibited significant antitumor activity in patients with HER2-expressing metastatic CRC, with a confirmed objective response rate of 45.3% in the DESTINY-CRC01 trial (3). In addition, there is evidence indicating that sacituzumab govitecan, a Trop-2-directed ADC, can achieve meaningful responses in refractory metastatic CRC, particularly in populations with limited therapeutic options (4). However, any claims regarding broad ADC efficacy in CRC must be approached with profound caution. As our landscape stratification reveals, the absolute majority of global trials remain in Phase I or Phase I/II development. Because these early-stage studies are fundamentally safety-oriented and often underpowered to detect definitive survival metrics, long-term survival benefits for the wider CRC population are yet to be definitively established. Furthermore, different ADC targets present highly divergent risk-benefit profiles. For instance, while HER2-targeted ADCs like T-DXd demonstrate high response rates, they are also associated with specific toxicities such as interstitial lung disease, necessitating close patient monitoring. In contrast, Trop-2-targeted ADCs like sacituzumab govitecan demonstrate broader applicability across heterogeneous tumor expression patterns, though they may be constrained by hematologic toxicity. Such variations underscore the necessity for target-specific risk-benefit assessments and the development of personalized management strategies.

Although the therapeutic potential of ADCs has been initially explored, current clinical research on ADC treatments for CRC faces multiple obstacles. While interpreting these landscape trends, it is essential to distinguish between data-driven findings and broader ecosystem hypotheses. First, significant publication bias is prevalent—positive results are more likely to be published in peer-reviewed journals or international conferences, while negative or neutral findings such as data from some early c-Met-targeted ADC trials often remain outside the academic sphere. This information asymmetry may distort the academic community’s overall assessment of ADC efficacy, thereby affecting the objectivity of clinical treatment decisions. Second, our data demonstrate a heavily industry-sponsored research landscape. This distribution naturally aligns with standard commercial development strategies that focus investment on established hotspot targets with validated biological mechanisms (e.g., HER2, Trop-2). While this clustering reflects rational risk mitigation and accelerates the clinical translation of verified therapeutics, it simultaneously results in a comparatively smaller volume of trials investigating emerging or niche targets in less common colorectal cancer subtypes (e.g., Claudin 18.2 and CEA). Balancing industry-driven optimization of validated pathways with exploratory research into novel biomarkers remains an ongoing evolutionary feature of the global ADC pipeline.Furthermore, the high cost of antibody-drug conjugates, complex access barriers including diagnostics and reimbursement, and the lack of clear cost-effectiveness evidence present systemic challenges that must be addressed during the transition from clinical trial results to widespread adoption. These factors pose significant obstacles to the long-term clinical application of ADCs in colorectal cancer patients.

Despite frequent ADC development activities, most projects remain in early clinical trials and struggle to advance to pivotal studies. The precise driving factors for trial discontinuation within specific sub-populations remain difficult to isolate systematically due to limited public disclosure. In broader oncology clinical pharmacology, pipeline attrition typically stems from a complex interplay of clinical safety, efficacy margins, and commercial viability. General structural hurdles in ADC design—such as tumor heterogeneity, complex resistance mechanisms, or unpredictable pharmacokinetic profiles—remain broad challenges that the academic and industrial fields continue to address through technological refinement; while the complex mechanisms of action and pharmacokinetics (PK) of ADCs often result in disproportionate drug concentrations between tumor tissue and plasma, where tumor uptake does not necessarily correlate with therapeutic efficacy. To address these challenges, research in this field primarily focuses on improving ADC design through optimized target selection, linker stability, and payload efficiency, aiming to enhance their therapeutic index.

It is imperative to highlight that the recent consensus guidelines from the NCCN have incorporated recommendations for biomarker-directed use of ADCs in select CRC subpopulations (5). Also, preclinical advances, such as novel conjugation technologies and immune-stimulating payloads, offer promising avenues to enhance the efficacy of ADCs. In addition, resistance mechanisms including payload efflux and antigen heterogeneity warrant further investigation to improve patient outcomes. Policy initiatives that incentivize research into resistance mechanisms and support combination therapy trials are essential to systematically address these barriers and translate scientific progress into meaningful patient benefit. Ultimately, because the current pipeline is predominantly comprised of early-stage, safety-driven trials, the therapeutic efficacy observed in select studies cannot yet be generalized as standard clinical recommendations.

## Conclusion

This analysis highlights the significant progress made in the research, development, and clinical application of ADCs for colorectal cancer treatment, while also revealing persistent early-stage bottlenecks. Future advances will rely on the discovery of novel molecular targets, optimization of linker stability and conjugation techniques, development of more potent and customizable payloads, and fine-tuning of antibody affinity to improve tumor penetration. Furthermore, rational combination strategies with immunotherapy, targeted agents, and tumor microenvironment modulators hold promise to amplify antitumor efficacy and durability. While this landscape-level synthesis provides an integrated view of how targets, payloads, sponsors, and regions shape the current ADC pipeline in colorectal cancer, it is inherently constrained by the early phase of most included trials, the absence of individual patient data, and a lack of formal cross-trial comparative statistics. Accordingly, the conclusions drawn here regarding therapeutic efficacy should be regarded as highly cautious and hypothesis-generating. This strategic guidance is intended for future trial design, rather than definitive evidence for regimen selection in routine clinical practice. The analysis can guide the prioritization of subsequent biomarker-driven trials, including those that will be adequately powered for formal comparative evaluation.

## Data Availability

The datasets generated and analyzed during the current study are available from the corresponding author upon reasonable request, in accordance with applicable ethical and institutional requirements.

## Supplementary material

Supplementary Figure S1 PRISMA-style flow diagram detailing the clinical trial selection process. Multi-cohort and basket trials were rigorously assessed, and only those with clearly extractable data for colorectal cancer (CRC) cohorts were retained. Duplicate entries across international registries were systematically identified and removed to ensure data integrity.
