# Supplementary figures and images for: Antibody-drug conjugates in colorectal cancer: current landscape and future perspectives from clinical trials
Source: Front Oncol. 2026 Jun 19;16:1843037. doi: 10.3389/fonc.2026.1843037 (PMC13327900; doi:10.3389/fonc.2026.1843037)

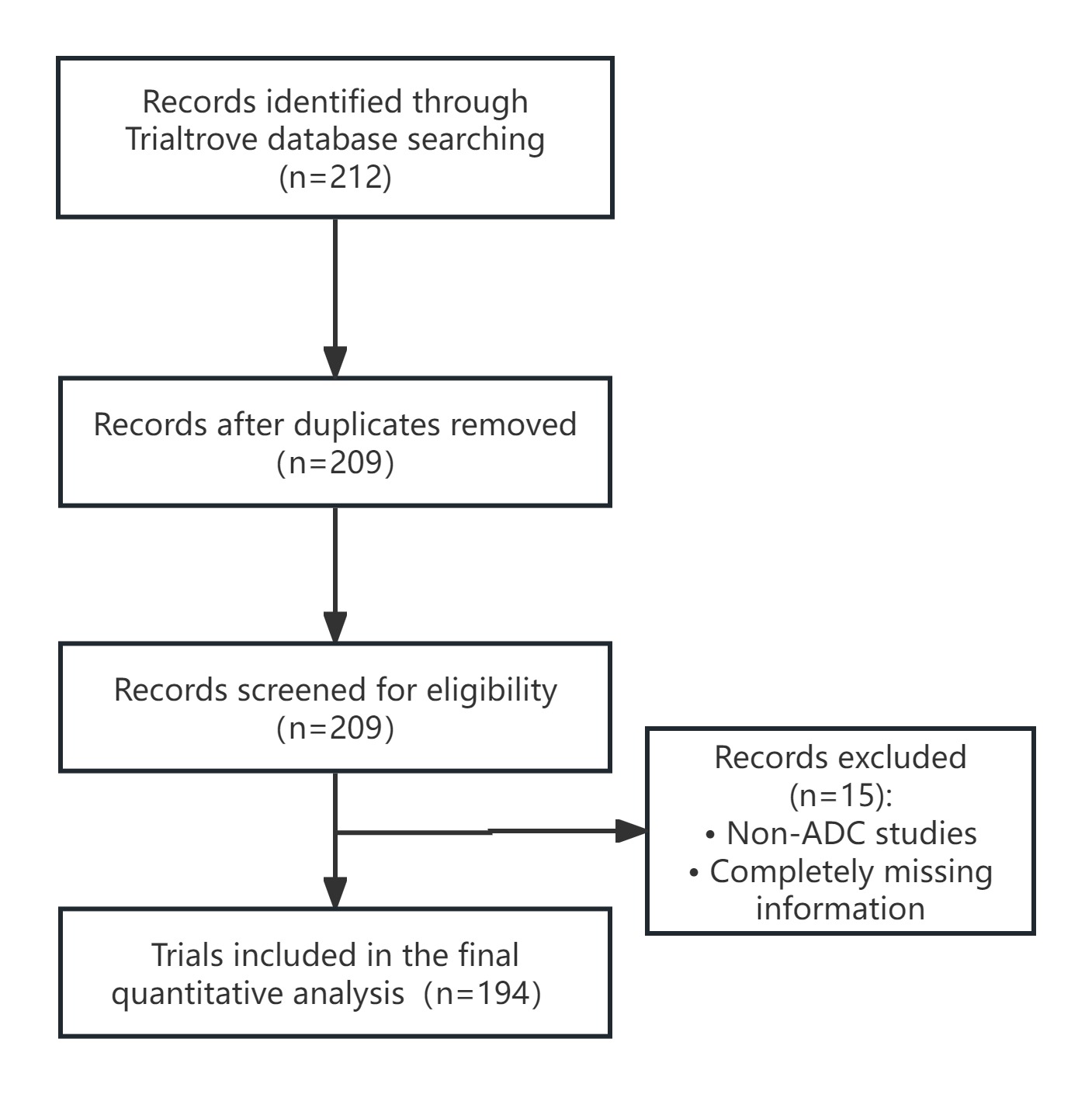

Supplement: Supplementary Figure S1 — PRISMA-style flow diagram detailing the clinical trial selection process. Multi-cohort and basket trials were rigorously assessed, and only those with clearly extractable data for colorectal cancer (CRC) cohorts were retained. Duplicate entries across international registries were systematically identified and removed to ensure data integrity. [file Image1.jpeg]
