# Supplementary material for: Antibody-drug conjugates in colorectal cancer: current landscape and future perspectives from clinical trials
Source: Front Oncol. 2026 Jun 19;16:1843037. doi: 10.3389/fonc.2026.1843037 (PMC13327900; doi:10.3389/fonc.2026.1843037)
Supplement: Supplementary file 3 [file Table1.docx]

**Supplementary Table S1. Classification criteria and definitions for clinical trial analytical indicators.**

| **Category** | **Classification / Subgroups** | **Definition and Inclusion Criteria** |
| --- | --- | --- |
| **Target Antigens** | HER2, Trop-2, c-Met, Claudin 18.2, CEA, Others | Classified based on the primary molecular target of the antibody component. "Others" includes niche or emerging targets (e.g., B7-H3, EGFR, AXL) with limited trial counts. |
| **Payload Mechanism** | Topoisomerase I inhibitors (TOPIs), Tubulin inhibitors, DNA damaging agents, Others | Categorized by the primary cytotoxic mechanism of action (MOA) of the payload. Dual-payload ADCs were classified based on their dominant or primary reported mechanism. |
| **Linker Type** | Cleavable, Non-cleavable, Undisclosed | Classified by the chemical release mechanism. Cleavable linkers depend on physiological conditions (e.g., pH, proteases) for payload release, facilitating the bystander effect. Non-cleavable require complete lysosomal degradation of the antibody. |
| **Trial Phase** | Phase I, Phase I/II, Phase II, Phase III, Phase IV | Based on the official registry designation. Early-stage trials were defined as Phase I and Phase I/II. Late-stage, pivotal trials were defined as Phase III and Phase IV. |
| **Sponsor Type** | Industry, Academic / Institutional, Government / Cooperative Groups | **Industry**: Trials fully or partially funded/sponsored by pharmaceutical or biotechnology companies.  **Academic/Government**: Investigator-initiated trials or those sponsored by universities, hospitals, or government entities without primary commercial funding. |
| **Trial Status** | Open/Recruiting, Active (not recruiting), Completed, Terminated/Suspended/Withdrawn | **Active/Recruiting**: Currently enrolling or treating patients.  **Completed**: Trial has reached primary completion.  **Terminated/Suspended**: Halted prematurely due to safety, lack of efficacy, or business decisions. |
